# Double Strike Approach for Tumor Attack: Engineering T Cells Using a CD40L:CD28 Chimeric Co-Stimulatory Switch Protein for Enhanced Tumor Targeting in Adoptive Cell Therapy

**DOI:** 10.3389/fimmu.2021.750478

**Published:** 2021-11-29

**Authors:** Luis Felipe Olguín-Contreras, Anna N. Mendler, Grzegorz Popowicz, Bin Hu, Elfriede Noessner

**Affiliations:** ^1^ Institute of Molecular Immunology, Helmholtz Center Munich, Munich, Germany; ^2^ Institute of Structural Biology, Helmholtz Center Munich, Munich, Germany; ^3^ Immunoanalytics Research Group - Tissue Control of Immunocytes, Helmholtz Center Munich, Munich, Germany

**Keywords:** co-stimulation, adoptive cell therapy, dendritic cell maturation, tumor microenvironment, immune therapy, chimeric switch protein, tumor stroma, CD40/CD40L

## Abstract

Activation of co-stimulatory pathways in cytotoxic T lymphocytes expressing chimeric antigen receptors (CARs) have proven to boost effector activity, tumor rejection and long-term T cell persistence. When using antigen-specific T cell receptors (TCR) instead of CARs, the lack of co-stimulatory signals hampers robust antitumoral response, hence limiting clinical efficacy. In solid tumors, tumor stroma poses an additional hurdle through hindrance of infiltration and active inhibition. Our project aimed at generating chimeric co-stimulatory switch proteins (CSP) consisting of intracellular co-stimulatory domains (ICD) fused to extracellular protein domains (ECD) for which ligands are expressed in solid tumors. The ECD of CD40L was selected for combination with the ICD from the CD28 protein. With this approach, it was expected to not only provide co-stimulation and strengthen the TCR signaling, but also, through the CD40L ECD, facilitate the activation of tumor-resident antigen-presenting cells (APCs), modulate activation of tumor endothelium and induce TCR-MHC independent apoptotic effect on tumor cells. Since CD28 and CD40L belong to different classes of transmembrane proteins (type I and type II, respectively), creating a chimeric protein presented a structural and functional challenge. We present solutions to this challenge describing different CSP formats that were successfully expressed in human T cells along with an antigen-specific TCR. The level of surface expression of the CSPs depended on their distinct design and the state of T cell activation. In particular, CSPs were upregulated by TCR stimulation and downregulated following interaction with CD40 on target cells. Ligation of the CSP in the context of TCR-stimulation modulated intracellular signaling cascades and led to improved TCR-induced cytokine secretion and cytotoxicity. Moreover, the CD40L ECD exhibited activity as evidenced by effective maturation and activation of B cells and DCs. CD40L:CD28 CSPs are a new type of switch proteins designed to exert dual beneficial antitumor effect by acting directly on the gene-modified T cells and simultaneously on tumor cells and tumor-supporting cells of the TME. The observed effects suggest that they constitute a promising tool to be included in the engineering process of T cells to endow them with complementary features for improved performance in the tumor milieu.

## Introduction

It is well known that the tumor microenvironment (TME) can induce antigen-specific tolerance or anergy by several different mechanisms (1). Restriction of T cell infiltration as well as inhibition of T cell functionality through nutrient depletion and accumulation of suppressive metabolites and regulatory cells are some of the mechanisms that shape an ineffective antitumor response with subsequent failure to control tumor growth (2). T cells used for adoptive cell therapy (ACT) are preselected for optimal antigen specificity and strong functional capacity. Still, they develop hyporesponsiveness once infiltrating the TME (3–6). T cells require support to perform effective cytolysis and cytokine secretion, to be able to proliferate and be protected from apoptosis. The field of synthetic biology has emerged as a means to combine elements of different disciplines, including engineering, chemistry, computer science and molecular biology, gathering necessary cellular and biological tools to improve the natural function of the T cells to be used for ACT (7). One promising strategy to provide T cells with necessary support signals is to provide a synthetically engineered co-stimulation (8). The power of engineered co-stimulation is evidenced in the use of chimeric antigen receptors (CARs), which was one of the first successful strategies to overcome the hampered T cell antitumor response. First generation CARs did not show the expected efficacy due to the failure of the genetically modified cells to expand and persist in the patient. However, when a co-stimulatory domain was integrated into the CAR design, T cell functionality and persistence improved significantly, evidencing the beneficial effect of this approach (9, 10).

TCR-engineered CD8 T cells have also been used in clinical trials (11), with efficacies, however, still remaining behind expectation (12). Considering the lack of CD28 expression on most human CD8 T effector cells, including those infused in patients (13, 14) and the observed positive association of CD28 expression with clinical benefit (15), facilitating co-stimulation is hypothesized to be one means towards improving efficacy. While in the CAR design the co-stimulatory sequence is fused in line with the antigen specificity and CD3zeta for TCR signaling, the co-stimulatory sequence cannot be fused to the antigen-specific TCR sequence because this will hinder TCR signaling. Moreover, reconstituting the T cells with the native CD28 co-stimulatory protein would not be advantageous in the milieu of most solid tumors, because CD28 ligands (CD80, CD86) that trigger the CD28 surface receptor function are generally absent. Therefore, chimeric co-stimulatory proteins are explored that utilize protein domains, which have cognate interaction partners in the TME, to activate co-stimulatory support for T cell function (16–19). Several designs are currently explored using the extracellular domain of an inhibitory receptor (CTLA-4, PD-1, TIGIT, CD200R, Fas) and linking it to an intracellular domain of a co-stimulatory protein like CD28, 4-1BB or OX40, thereby not only preventing the inhibitory signal but additionally switching T cell inhibition signals into T cell activation upon engagement with the inhibitory ligand (20–25).

While co-stimulation can enhance TCR signaling and effector function as well as extend the operative life span of CTLs (26–28), T cells in solid tumors additionally face the tumor stroma that not only hinders infiltration but also actively mediates inhibition (29).

**Figure 1:**
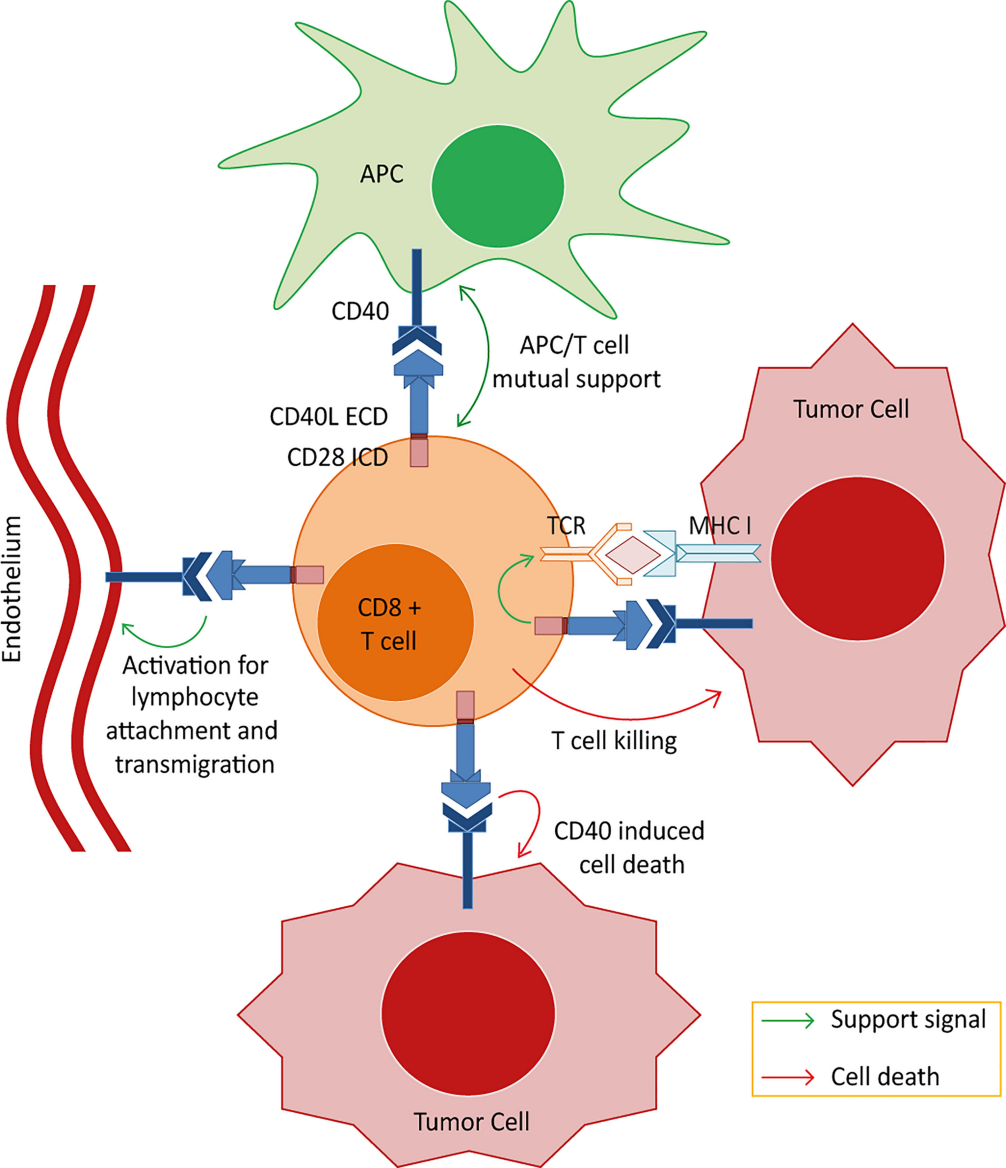
Postulated effector mechanisms for the CD40L:CD28 chimeric co-stimulatory switch proteins (CSPs). Depicted are the 4 potential axes of effector mechanisms of T cells engineered with the CD40L:CD28 CSPs when interacting with different components of the tumor microenvironment. 1) Strengthening the MHC/peptide-triggered TCR signaling through eliciting an intracellular co-stimulatory signaling cascade allowing the T cells to overcome inhibition of effector function in the tumor milieu. 2) Activation of tumor-resident CD40-expressing DCs. Interaction of CD40 on DCs with CD40L expressed on the engineered T cells could induce signals in DCs leading to their maturation with gain in *de novo* priming capability. 3) Targeting tumor endothelium. CD40 is expressed, amongst others, on neovascular endothelium and CD40 stimulation has been shown to activate human endothelial cells including proliferation and the upregulation of adhesion molecules, enabling T cell attachment and infiltration. Targeting this stromal compartment could potentially enhance the immunotherapy effect by depriving the tumor bed of live supporting surroundings and enhancing T cell infiltration. 4) Apoptotic effects on tumor cells. It is reported that tumor cells aberrantly express CD40 and that CD40 signals induce apoptotic cell death independent of MHC/peptide-specific targeting. Thus, CD40L:CD28-engineered T cells may kill tumor cells expressing CD40 even if they do not present the cognate TCR-MHC ligand.

Our project aimed at generating a novel chimeric co-stimulatory switch protein (CSP) consisting of an intracellular co-stimulatory domains (ICD) fused to the extracellular domains (ECD) of a protein that utilizes ligands expressed in the tumor milieu. Such CSP should mediate antitumor effects on different levels including stromal attack and enhanced antitumoral T cell activity. CD40L (CD154) was selected as the donor protein for the ECD because of the pivotal role that the CD40/CD40L pathway plays in humoral and cellular immunity (30, 31). Moreover, the CD40L interaction partner (CD40) is available in the TME, being aberrantly expressed not only on a wide variety of carcinoma cells (32–35) but also on other cells in the TME, such as antigen presenting cells (APCs) and endothelium (36–42). Thus, CD40L:CD28 CSPs can be triggered in the TME and should, thereby, activate the co-stimulation in CD40L:CD28-engineered T cells while additionally supporting the antitumor response indirectly by inducing tumor cell apoptosis (43, 44), and counteracting various suppressive mechanisms of tumor-stroma components, including immune stimulatory polarization of DCs and tumor-associated macrophages (45–48), counteracting T regulatory cells (49), and modulating the tumor endothelium for improved T cell infiltration (40, 42) (**Figure 1**). In this way, the CD40L:CD28 CSPs should differ to other switch receptors, such as PD1:CD28, CTLA4:CD28 and anti-TGFβ:CD28, which exert their effects mainly on the expressing T cells (50).

We report here the molecular design of novel CD40L:CD28 CSPs and the functional activity of CD40L:CD28 CSP-engineered TCR-transgenic (tgTCR) T cells. Augmented T cell activity as well as DC maturation and induction of a pro-inflammatory secretome of tumor-conditioned DCs are demonstrated. CD40L:CD28 CSPs were observed to display a dynamic surface expression dependent on T cell activation and CD40 interaction. They delivered co-stimulatory signals and functional support to the T cells in a timely manner when the T cell interacted with the tumor cell and for as long as the tumor cell was present. Collectively, the concept of integrating T cell co-stimulation with the pathways that the CD40/CD40L interaction offers to support the immune response into a chimeric CD40L:CD28 design is a very promising approach to provide multiple key upgrades to T cells for enhanced efficacy in adoptive T cell therapy.

## Materials and Methods

### Primary Cells and Cell Lines

Human PBMC from healthy donors were isolated by ficoll density gradient centrifugation and used for CSP transduction, isolation of B cells and monocytes. The blood collection was done with approval by the institutional review board of Ludwig-Maximilians-University, Munich, Germany. Donors gave informed consent in accordance with the Declaration of Helsinki. PBMC used for B cell isolation and DC generation were from HLA-A2 negative donors such that the B cells or DCs did not provide antigen ligands for the TCR-T58, TCR-D115 or TCR53- tgTCR-T cells in co-culture experiments.

Primary B cells were isolated from PBMC using the CD19 magnetic (negative isolation) Naïve B Cell Isolation Kit II, human (Miltenyi Biotec, Bergisch Gladbach Germany), according to the manufacturer´s instruction. Primary B cells were used freshly prepared for the B cell activation assay. Monocytes were derived from PBMC by plastic adherence and were differentiated to immature dendritic (iDCs) by adding 100 ng/ml GM-CSF and 20 ng/ml IL-4 (both Promokine, PromoCell, Heidelberg, Germany) to VLE RPMI-1640 supplemented with 1% L-glutamine, 1% non-essential amino acids, 1% sodium pyruvate, 1% penicillin/streptomycin and 5% human serum (DC-medium) on day 1 and day 3. iDC were obtained at day 7 and used for co-culture with T cells. Mature DC (mDC) were generated from day7 iDCs by adding Jonuleit maturation cocktail (20 ng/ml IL-4 (PromoCell), 100 ng/ml GM-CSF (PromoCell), 15 ng/ml IL-6 (Sigma-Aldrich), 10 ng/ml IL-1β (PromoCell), 20 ng/ml TNFα (Immunotools) and 1 µg/ml PGE_2_ (PromoCell) for 24 h (51).

Monocytes were used to generate ercDC (*enriched in renal cell carcinoma*) DCs as described (52). Briefly, monocytes from human PBMC were cultivated in DC medium supplemented with 20% RCC-26 conditioned medium on days 1, 3 and 5 for 7 days. RCC-conditioned medium was derived from 2 x 10^6^ renal carcinoma cell line RCC-26 cultivated for 10 days in serum-free AIM-V medium (Gibco/Invitrogen, Carlsbad, CA), then centrifuged and filtered to remove cells (52).

T cells used for retroviral transduction were either primary human T cells from PBMC or human T cells expressing transgenic TCRs (tgTCR)-T cells either with HLA-A2 restricted specificity for the tyrosinase peptide (TCR-T58 or TCR-D115) (53) or with HLA-A2 restricted renal cell carcinoma (RCC) specificity (TCR53) (19, 54). T cells were cultured in RPMI1640 supplemented with 1% L-glutamine, 1% non-essential amino acids, 1% sodium pyruvate, 1% penicillin/streptomycin (RPMI-basic) and 10% human serum (T cell medium, TCM) with human IL-2 (Cancernova GmbH, Reute, Germany) at concentration as indicated.

Cell lines were the human melanoma line SK-Mel23 (ATCC HTB71, from M.C. Panelli, NIH/Bethesda, USA), the human RCC cell lines RCC-53 and RCC-26 (isolated from RCC tissue in our laboratory) (54), the HEK293/Tyr cells transduced in our laboratory to express tyrosinase (HEK/Tyr) (19) and HEK293/Tyr/CD40, which are HEK293/Tyr cells subsequently transduced to express CD40. For HEK293/Tyr and HEK293/Tyr/CD40 single-cell clones were selected for comparable HLA-A2 and tyrosinase expression. HEK293 cells with stable expression of CD40L (HEK293/CD40L) were kindly provided by Kathrin Gärtner, Helmholtz Center Munich. All cell lines were grown in RMPI-basic with 10% FCS at 37°C/6.5% CO2. Mycoplasma testing was performed monthly using VenorGeM Classic (Minerva biolabs, Germany). All cell lines were authenticated by flow cytometry to express the relevant molecules, which were HLA-A2, tyrosinase, CD40, or CD40L, as required.

### CD40L:CD28 CSP Constructs and Retroviral Transduction

Three different CD40L:CD28 CSP constructs were designed. Two of them created a type I membrane protein structure in which the C-terminal part corresponding to the soluble CD40L fragment (AA 113-261) was inverted and linked to the transmembrane (TMD) plus cytoplasmic domain (ICD) of CD28 (AA 153-220). For successful intracellular trafficking and surface expression, the PD-1 signal peptide (20 AA) was used. ECD and ICD were connected through a Glycine/Serine linker (10 AA), which provides flexibility of the connected functional domains, and a spacer, either IgG1Fc or Fil3 (third Ig-like repetition corresponding to the Filamin protein), for expression improvement. These CSPs were designated as CD40L:IgGFc:CD28 and CD40L:Fil3:CD28, respectively. The third construct adopted a type II transmembrane protein structure by linking the CD40L ECD and TMD (AA 14-261) with the inverted ICD (AA 180-220) of CD28. This construct was designated as CD40L:CD28i. The CD40L native sequence (provided by Kathrin Gärtner, Helmholtz Center Munich, Germany) and the chimeric sequences (ordered from GeneArt) were cloned in the pMP71 vector for retroviral transduction of primary T cells (CD3/CD28-activated PBMC), or tgTCR-T cells expressing TCR-T58, TCR-D115 or TCR53. Retroviral transduction was performed as described (19, 55). Briefly, human PBMC or tgTCR-T cells were thawed and activated with 5 μg/ml of plate-bound OKT3 (provided by E. Kremmer, Helmholtz Center Munich, Germany) and 1 μg/ml of anti-CD28 (BD Bioscience) for 2 days in TCM with 100 U/ml IL-2. Thereafter, T cells were split into 5 equal parts, each transduced with retrovirus particles encoding either the native CD40L sequence or one of the three chimeric CD40L:CD28 constructs, or no construct (tgTCR/Mock-T cells). After 4 days, CSP-transduced T cells were harvested and cultivated for another 12 days reducing the amount of IL-2 to 50 U/ml. CSP-transduced T cells were frozen on day 12 after transduction.

### Re-Activation of T Cells to Induce CD40L:CD28 CSP Surface Expression

For re-activation of T cells to induce CD40L:CD28 CSP surface expression, T cells, which were frozen on day 12 after CSP transduction, were thawed and stimulated either with anti-CD3 plus anti-CD28 antibodies or through target cell-expressed peptide/MHC TCR ligands.

For anti-CD3/CD28 stimulation, CD40L:CD28 CSP-transduced T cells were thawed and seeded in 24-well non-tissue culture treated plates that had been coated with anti-CD3 (OKT3, 5 µg/ml) and anti-CD28 antibodies (1 µg/ml) (1 x 10^6^ T cells/ml TCM with 100 U/ml IL-2). After 3 days, T cells were removed from the stimulation plate, diluted 1:4 and further cultured without anti-CD3/CD28 antibodies in TCM with 100 U/ml IL-2 for 3 additional days. On day 6, when endogenous CD40L was downregulated (evidenced by absence on CD4 Mock T cells by flow cytometry) and CSPs were still expressed, T cells were harvested and prepared for flow cytometry or used in B cell and DC activation assay.

For re-activation through peptide/MHC ligands, TCR-T58 or TCR-D115 T cells co-expressing the CD40L:CD28 CSPs or native CD40L, or Mock, were thawed and co-cultured with target cells at a ratio of 1:10. Target cells were HEK293/Tyr cells that had very low endogenous CD40 expression or HEK293/Tyr/CD40 that were transduced to strongly express CD40; or the melanoma cell line SK-Mel23 that endogenously expresses CD40. SK-Mel23 was used either untreated or was pretreated with anti-CD40 antibody (clone HB14 pure functional grade, Miltenyi, at 1:11 concentration) before setting up the co-culture to block the endogenous CD40. After 24 h co-culture, cells were harvested for flow cytometry.

### Flow Cytometry for CD40L:CD28 CSP Expression and Target Cell Characterization

To analyze surface expression of CD40L or CD40L:CD28 CSP, anti-CD40L-PE (89-76, eBioscience) was used in combination with anti-mouse TCRβ-constant region (mTCR)-PB (H57-59, BioLegend) to detect the transgenic TCR expression, anti-CD4-APC-Cy7 (RPA-T4, eBioscience) and anti-CD8-V500 (RPA-T8, BD). 7-AAD (Sigma-Aldrich) was used for live/dead discrimination. HLA-A2 and CD40 expression on HEK293/Tyr cells, HEK393/Tyr/CD40 cells and tumor lines SK-Mel23, RCC-26 and RCC-53 were analyzed using anti-HLA-A2 (ATCC HB54) plus anti-mouse IgG1-A488 (Invitrogen) and anti-CD40-PE (clone H-10, Santa Cruz Biotechnology) together with 7-AAD. Flow cytometry was performed with the LSRII (BD) cytometer and FlowJo v10.7.1 software.

### T Cell Stimulation and Flow Cytometry to Investigate Phosphorylated Signaling Proteins

TCRtg-T cells transduced with CD40L:CD28 CSPs were thawed and activated for 6 days using anti-CD3 and anti-CD28 antibody coated plates to upregulate the surface expression of the CD40L:CD28 CSPs (see Re-Activation of T Cells). On day 6, when the endogenous CD40L was downregulated on T cells and CD40L:CD28 CSPs were still expressed, T cells were harvested and washed with PBS, then incubated for 4 h in RPMI-basic without IL-2 to reduce constitutive background p-AKT signals. After 4 h, T cells were harvested, washed with PBS/EDTA (2 mM), and stained with fixable Blue reagent (Thermo Fisher Scientific/Caltag, WalthamMassachusetts USA) for viability (10 min), then washed and co-cultured with HEK293/Tyr/CD40 (1:2 ratio) for 30 min at 37°C and 5% CO_2_ to stimulate the T cells through the tgTCR-Tyr/HLA-A2 interaction and to trigger the CSPs through CD40. Unstimulated T cells were kept in culture as a negative control. After 30 min, co-cultures were immediately fixed using Cytofix Fixation Buffer (BD) (15 min, 37°C) followed by permeabilization with ice cold Phosflow Perm Buffer III (BDPhosflow™, 30 min). Antibodies to surface markers, anti-CD45-PE-Cy7 (HI30, BioLegend), anti-CD3-PerCP-Cy5.5 (SK7, eBiosciences), were added together with antibodies for the phosphorylated intracellular proteins p-AKT-PB (S473, M89-61, BD Biosciences), p-mTOR-A647 (S2448, O21-404, BD Biosciences) and p-RPS6-PE (pS235/pS236, (N5-676, eBiosciences). Data were acquired on the LSRII cytometer and analyzed using the FlowJo v10.7.1 software.

### B Cell Activation Assay

T cells transduced with CSPs and re-activated using anti-CD3 plus anti-CD28 antibody-coated 24-well plates (as described in Re-Activation of T Cells) were co-cultured at a 1:1 ratio with naïve human B cells per triplicate in 96-well U-bottom plate overnight at 37°C/5% CO_2_. Naïve B cells without stimulation were used as negative control. Positive controls were B cells activated using soluble enhanced trimeric CD40L reagent (Enzo Life Science) (1:10 dilution) and B cells activated using HEK293/CD40L cells. After overnight incubation, cultures were harvested and analyzed by flow cytometry for B cell specific surface activation markers CD83-PE (HB15a, Immunotech), CD86-FITC (2331, BD Biosciences) and Fas-PE-Cy7 (DX2, BioLegend) together with CD19-A700 (HIB19, BD Biosciences) and 7-AAD.

### DC Maturation Assay

iDCs (0.1 x 10^6^ cells) or ercDCs were harvested and co-cultured with T cells re-activated to express CD40L:CD28 CSPs (see Re-Activation of T Cells) at 1:1 ratios in 96-well U-bottom plates in a final volume of 200 µl TCM. Co-cultures were set in triplicates. iDCs alone, mDC, and iDCs with mock T cells were used as controls. After 24 h, supernatants were harvested for 45Plex bead array and cells were used for flow cytometry using antibodies to CD83-PE (HB15a, Immunotech), CCR7-PB (G043H7, BioLegend), PD-L1-PerCP-Cy5.5 (29E.2A3, BioLegend), HLA-DR-APC-Cy7 (L243, BD Biosciences) and CD80-PE-Cy7 (2D10, BioLegend) together with CD3-A700 (UCHT1, BioLegend) and live/dead fixable blue stain (Thermo Fisher Scientific/Caltag).

### Stimulation of T Cell Cytokine Secretion

TCRtg-T cells (TCR-T58, TCR-D115, TCR53) with or without co-expression of the different CSPs were thawed and co-cultured without re-activation with SK-Mel23, RCC-26 or RCC-53 at 1:2 T cell to target cell ratio in a 96 wells. T cells and target cells cultured alone were used as controls. All target cells expressed CD40 endogenously. Supernatants were collected after 24 h and analyzed for IFN-γ secretion by ELISA.

### T Cell-Mediated Target Cell Lysis Using Chromium Release Assay

T cell-mediated target cell killing was performed using a ^51^chromium release assay as described (56). Briefly, 1 x 10^6^ target cells were labeled with 50 μCi ^51^chromium (Hartmann Analytic, Braunschweig Germany) for 1 h at 37°C. TCRtg-T cells (TCR-T58, TCR-D115, TCR53) with or without the co-expression of the different CSPs were thawed and plated without re-activation at titrated cell numbers reaching T cell to target cell ratios from 10:1 to 1.25:1 in a 96 well plate. Chromium-labelled targets cells were added at a concentration of 2000 cells per well. For spontaneous release, target cells were cultured without T cells. Each culture was set up in duplicates. Cells were co-cultured for 4 h at 37°C. For maximum ^51^chromium release, 50 µl of the target cell suspension was pipetted directly to the Luma plate (Canberra Packard, Germany). After the co-culture time, 50 µl aliquot from each co-culture well was transferred to the Luma filter plate, dried and counted using a TopCount machine. Specific cell lysis was calculated by applying the formula:


$$\%\;cell\;lysis=\frac{\text{experimental}{\;}^{51}\text{Cr}-\text{release}-\text{spontaneous}{\;}^{51}\text{Cr}-\text{release}}{(\max.{\;}^{51}\text{Cr}-\text{release}/2)-\text{spontaneous}{\;}^{51}\text{Cr}-\text{release}}\times 100$$

### ELISA and Multiplex Bead Array

IFN-γ analysis of T cell/tumor cell co-cultures was done using ELISA kits (BD Bioscience) according to the manufacturer´s instructions. Supernatants of T cell/DC co-cultures were analyzed using a multiplex bead array 45Plex (LKTM014, human XL Cytokine, RnD Systems) according to the manufacturer’s instructions. Data were acquired using the Luminex100 machine with BioPlex Manager 6.1 software (Bio-Rad Laboratories GmbH). Standard curves were fitted using the logistic-5PL regression type.

## Results

### CD40L:CD28 CSP Design

**Figure 2:**
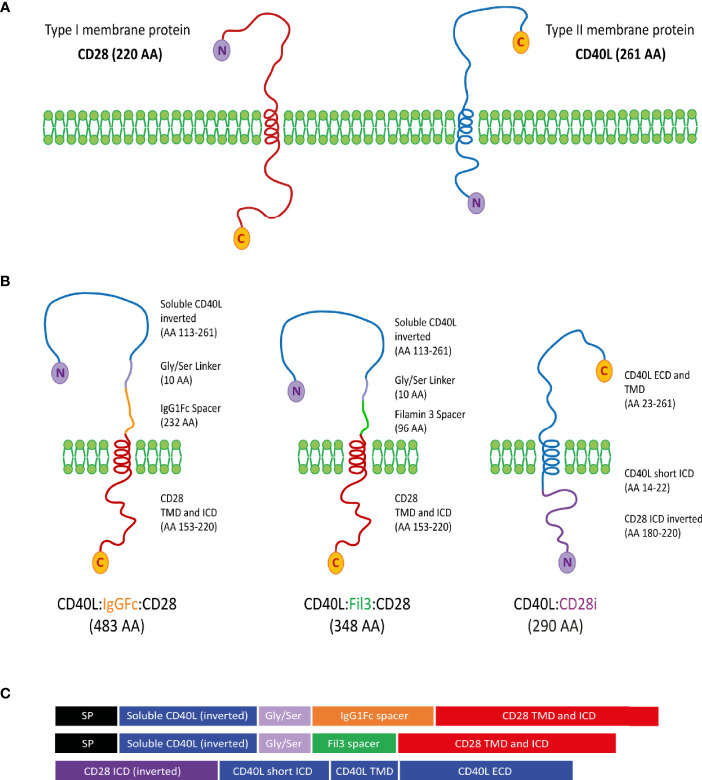
Design of CD40L:CD28 CSPs. **(A)** CD28 and CD40L are depicted according to their classification as type I and type II membrane proteins, respectively. C-terminal amino acid (C) and N-terminal amino acid (N) determines the membrane orientation. **(B)** Schematic representation of the three different approaches for the structure of the CD40L:CD28 CSPs, AA length and origin of protein fragments are specified next to each molecule. **(C)** Domain representation of the CSPs including the signal peptide (SP) from the PD-1 protein for type I membrane protein approaches. SP, signal peptide from PD-1 (20 AA); ICD, intracellular domain; TMD, Transmembrane domain; ECD, extracellular domain.

According to the topological classification of transmembrane proteins, the CD28 protein and the CD40L protein belong to the type I and type II groups, respectively (57, 58). A type I membrane protein is present on the cell surface with its N-terminus oriented towards the extracellular space and the C-terminus located on the cytoplasmic side. A type II membrane protein is anchored with a signal-anchor sequence and, once transported to the cell membrane, is located at the cell surface with its C-terminus oriented into the extracellular space and the N-terminus on the cytoplasmic side. With CD28 and CD40L having different membrane orientation, creating a chimeric CD40L:CD28 protein represented a structural and functional challenge (**Figure 2A**). Three different constructs were designed (**Figures 2B, C**). CD40L physiologically exists also as a soluble protein (AA 113-261) (59). Thus, it was considered that this CD40L ECD sequence should maintain functionality as inverted nucleotide sequence allowing to generate a type I protein structure with the transmembrane (TMD) and intracellular domain (ICD) sequences of CD28. The CD40L ECD and CD28 TM_ICD domains were connected through a Glycine/Serine linker for flexibility and mobility of the connected functional domains (60) and a specific spacer for expression improvement. The CSP variant CD40L:IgGFc:CD28 used the IgG1Fc domain as a spacer to provide the protein with a better membrane stability due to its dimerization property, which was observed during its previous use in the design of CARs for antigen-specific T cell engineering (61). In the second variant, CD40L:Fil3:CD28, the IgG1Fc spacer was exchanged for the third Ig-like repetition corresponding to the Filamin protein. This is a protein sequence that adopts an immunoglobulin-like fold without dimerization properties (62, 63). The rational for this variation was the concern of linking the primarily trimeric CD40L molecule with a dimerizing sequence like the IgG1Fc spacer. The potential oligomerization might result in the formation of protein clusters in the membrane that might interfere with the proper protein function. In the third construct, CD40L:CD28i, the type II transmembrane protein structure of the native CD40L was maintained linking the CD40L ECD plus TMD sequence to the inverted ICD sequence (AA 180-220) of CD28 (CD28i). This design followed that of a published NKG2D/CD3zeta chimeric protein (64). Sequences of the CSPs were cloned into pMP71 retroviral vector and used to stably transduce human T cells, which stably expressed either the HLA-A2 restricted tyrosinase-specific TCR, T58 or D115 (19, 53), or TCR53 (19, 54) specific for RCC.

### CD40L:CD28 CSP Surface Expression Efficacy Is Dependent on the CSP Design and Is Related to T Cell Activation

**Figure 3:**
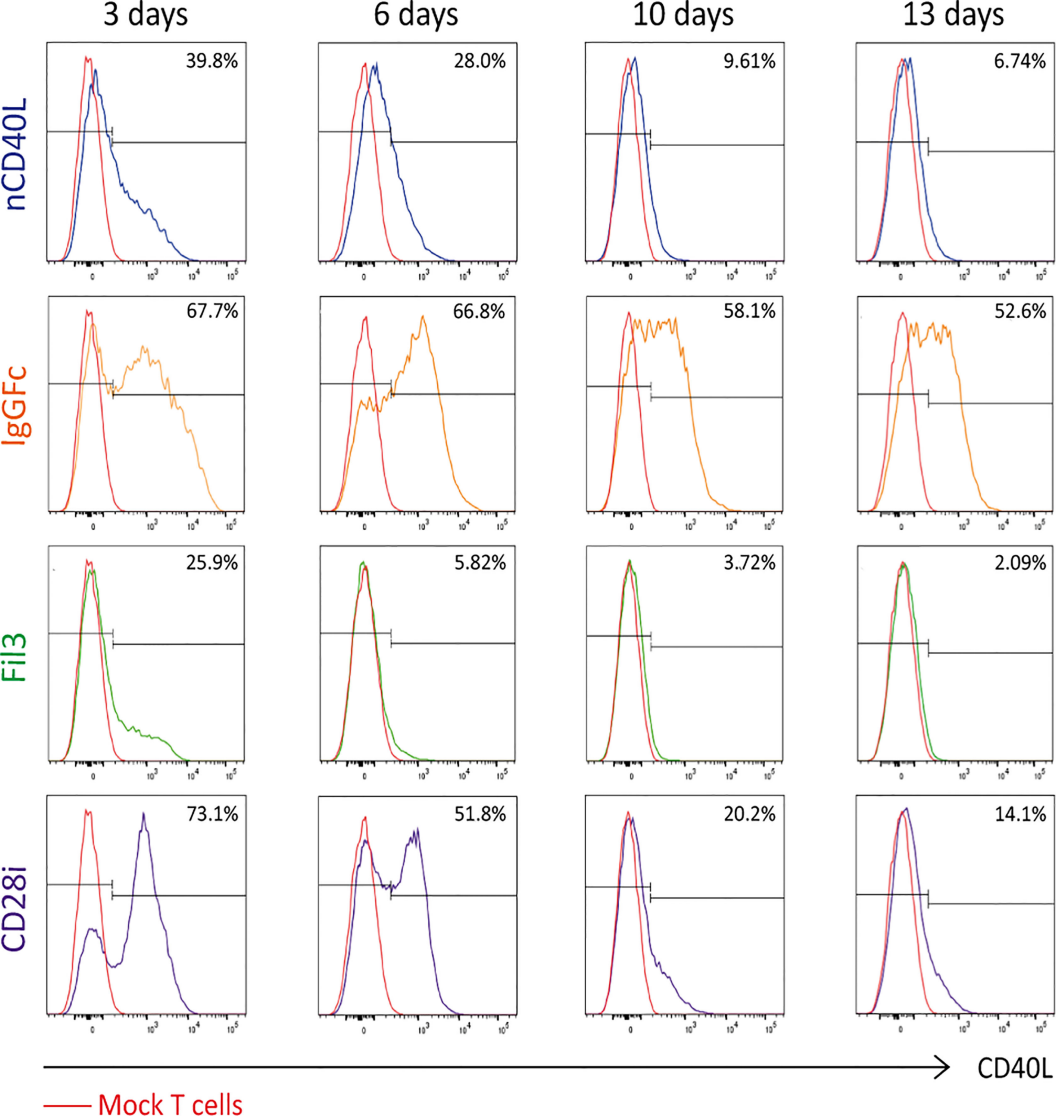
CD40L:CD28 CSP surface expression kinetic in PBLs after retroviral transduction. Human primary T cells were retrovirally transduced with pMP71 encoding the CD40L:CD28 sequences and CSP surface expression was measured by flow cytometry at 3, 6, 10 and 13 days after transduction using CD40L antibody. Percentage of CD40L-positive cells within gated live, single, CD3^+^ populations are displayed as histograms. Mock-transduced T cells were used as negative control (red line), T cells transduced with the native CD40L (nCD40L) protein were used as expression reference and are depicted in blue, CD40L:IgGFc:CD28 CSP is depicted in orange, CD40L:Fil3:CD28 CSP is depicted in green and CD40L:CD28i CSP is depicted in purple. Shown is one representative experiment of at least 5 repeats. A summary graph of 5 experiments is shown in **Supplementary Figure 1**.

**Figure 4:**
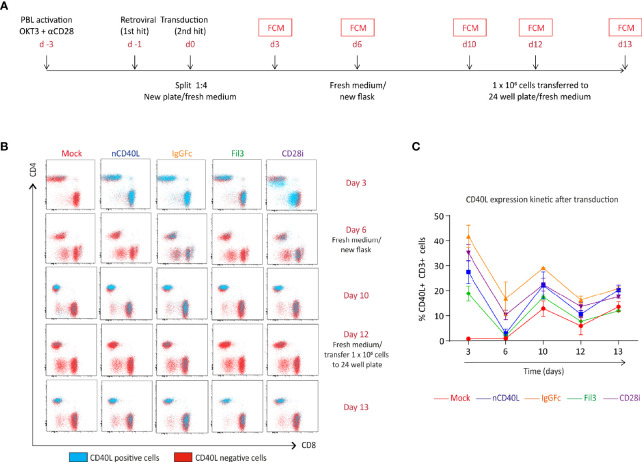
CD40L:CD28 CSP expression kinetic in T cells is modulated by medium replacement. Human primary T cells were retrovirally transduced with the native CD40L sequence or the CSP constructs and expanded for 12 days. Cells were transferred to a new flask or plate with fresh medium/100 U/ml IL-2 on day 6 and 12. **(A)** Timeline describing the transduction steps and expansion of human primary T cells. **(B)** CSP surface expression was measured by flow cytometry (FCM) at day 3, 6, 10, 12 and 13 after transduction using CD40L antibody. The CD4 and CD8 cells within the live, single, CD3^+^ population are depicted in the dot plots and the CD40L-positive (blue dots) and CD40L-negative (red dots) are graphically superimposed. Mock-transduced T cells were used as negative control and T cells transduced with the native CD40L (nCD40L) sequence were used as positive reference. **(C)** Percentage of CD40L-positive T cells at the different time points after transduction. Summary of 2 independent experiments, plus SEM. Mock-transduced T cells (red line) and T cells transduced to express the native CD40L (blue line) were used as reference. T cells transduced with CD40L:IgGFc:CD28 are depicted in orange, those with CD40L:Fil3:CD28 CSP are depicted in green and T cells with CD40L:CD28i CSP are depicted in purple.

To assess if the chimeric sequences are transcribed and translated correctly and are able to generate correctly folded proteins, which traffic to the cell surface, CSP-transduced T cells were stained with anti-CD40L antibody at different time points after transduction (3 days, 6 days, 10 days and 13 days) to detect the CSP on the cell surface. T cells transduced with the native sequence of CD40L (nCD40L) were used as control. It was observed that the expression profile varied depending on the CSP design (**Figure 3** and **Supplementary Figure 1**): at 3 days after transduction, the CD40L:IgGFc:CD28 and CD40L:CD28i showed highest expression with 67.7% or 73% CSP positive T cells, while the CD40L:Fil3:CD28 reached only 26% positive T cells. The expression decreased over time, for CD40L:IgGFc:CD28 from 76.7% to 52.6% at day 13, for CD40L:CD28i from 73.% to 14% and to undetectable levels for CD40L:Fil3:CD28. The loss of surface expression was also observed for the nCD40L indicating that it was not a feature of the chimeric proteins. Retroviral vectors should integrate into the genome and result in stable transgene expression. Thus, the loss of CD40L:CD28 CSP expression was surprising and unique to CD40L proteins, since it was not observed for other transgenes (TCRs, PD-1:CD28 CSP) expressed using the same retroviral vector pMP71 (**Supplementary Figure 2**). Cell culture conditions, as schematically depicted in **Figure 4A**, were found to relate to cell surface expression of the CD40L proteins (**Figures 4B, C**). Exemplary shown is cell splitting (density reduction) with addition of fresh medium containing IL-2, which was done on day 6 and day 12. Reproducibly, the splitting event turned nearly undetectable CSP expression (day 6, or day 12) on CD8 T cells to fair surface expression on day 10 or day 13 (**Figures 4B, C**: CSP positive cells are depicted in blue and are superimposed on CD40L negative cells depicted in red). A similar oscillation of expression was observed for the transgenically expressed native CD40L protein, suggesting that the CD40L extracellular domain, which is shared between CSP and native CD40L, might be involved in the expression kinetic. Indeed, it is well known that the endogenous expression of CD40L is tightly linked to T cell activation showing fast upregulation after stimulation and decay within 16-24 hours after stimulation. The natural kinetic is presumed to minimize bystander activation of CD40 positive cells (65). The kinetic of the endogenous CD40L expression in comparison to the kinetic of the CSPs is best observed in the dot plots of the mock transduced T cells (**Figure 4B**, Mock) within the CD4 T cell subset, which are known to express CD40L upon activation (66). In **Figure 4C**, a line graph depicts the surface expression kinetic of the CD40L:CD28 CSPs and the CD40L native protein upon changes in culture conditions on day 6 and day 12.

**Figure 5:**
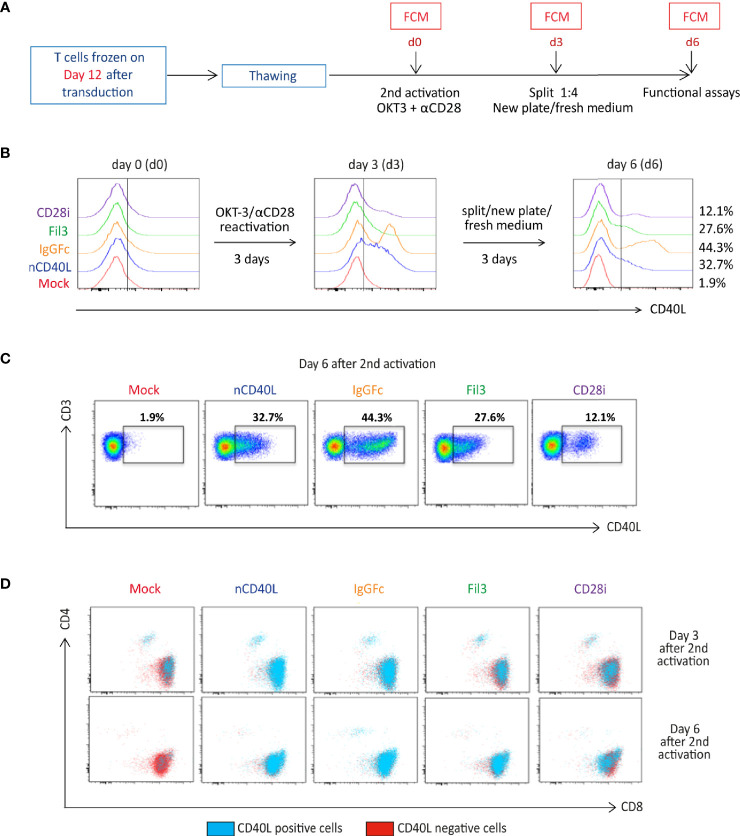
CD40L:CD28 CSP surface expression is regulated by anti-CD3/CD28 T cell activation. **(A)** Timeline depicting the re-activation of transduced T cells, which were frozen on day 12 after CSP transduction. T cells were thawed and re-activated using anti-CD3 plus anti-CD28 antibodies and 100 U/ml IL-2 for 3 days, then transferred to a new plate without antibodies in fresh medium plus 100 U/ml IL-2 for 3 additional days to allow downregulation of endogenous CD40L expression on Mock-T cells. **(B)** Histograms showing CD40L surface expression (identifying the CD40L:CD28 CSPs, the transduced native CD40L protein and the endogenously expressed CD40L) by flow cytometry after thawing and every third day after re-activation. **(C)** Gated CD40L-positive CD4 and CD8 cells within the live, single, CD3^+^ population are shown in dot plots as the blue population superimposed on the CD40L-negative T cells shown in red. Mock-transduced T cells were used as negative control and T cells transduced with the native CD40L (nCD40L) sequence are the reference against the CD40L:CD28 CSPs. **(D)** Percentage of live, single, CD3 positive cells are shown in dot plots measured 6 days after re-activation of CD40L:CD28 CSP-transduced T cells. This stimulation was performed for each B cell and DC assay, as well as CD28 signaling assay, thus at least 10 times. The exact number of repeats is listed with each of these processes.

Considering that the native CD40L expression is linked to T cell activation, it was evaluated if the CD40L:CD28 CSP expression might follow a similar behavior. To this end, CD40L:CD28 CSP-transduced T cells, which had been frozen on day 12 when CSP expression was lowest, were thawed and objected to an *in vitro* activation (**Figure 5A**) using anti-CD3 and anti-CD28 antibodies. On day 3, the activation signal was removed and activated T cells were cultured with fresh medium plus IL-2 for another 3 days. CD40L staining was performed to detect surface expression of the CD40L:CD28 CSPs directly after thawing (day 0), on day 3, and day 6. Depicted by histogram display (**Figure 5B**), it is showed that on day 0 CD40L:CD28 CSP expression is not detectable. On day 3 after activation, T cells had upregulated the expression of the CD40L:CD28 CSPs. Once the T cells were transferred to a new plate without antibody stimulation, the expression started to decline again, with variable percentages of expression remaining on day 6 depending on each construct (**Figures 5B, C**). The endogenous CD40L expression kinetic could be followed on the CD4 T cell population by dot plot display (**Figure 5D**) showing the loss of CD40L as evidenced by the absence of CD40L staining on CD4 mock T cells on day 6. Therefore, CD4 mock-transduced T cells were used as a negative control in further assays that assessed the CD40L:CD28 CSP functionality.

### CD40L:CD28 CSPs Are Upregulated by Peptide/MHC-TCR Stimulation and Downregulated by Interaction With the CD40 Receptor

**Figure 6:**
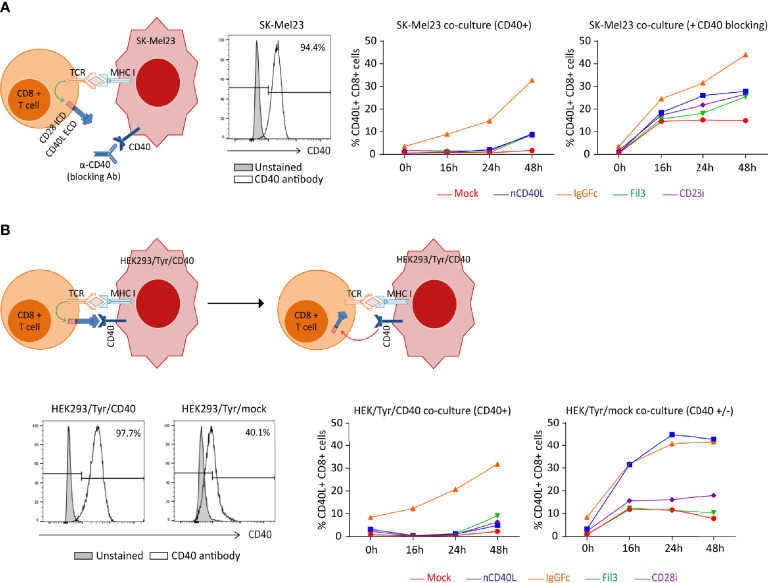
CD40L:CD28 CSP expression kinetic on T cells after recognition of peptide/MHC ligands on target cells in the presence or absence of target cell-expressed CD40 receptor. Human TCR-T58 T cells (tyrosinase-specific, HLA-A2 restricted) expressing the CD40L:CD28 CSPs were frozen on day 12 after CSP transduction. After thawing, T cells were used in co-cultures at a T cell to target cell ratio of 1:10. CSP surface expression on TCR-T58 T cells was analyzed by flow cytometry and is expressed as percentage of gated single, live, CD8+ CD40L+ cells. Mock-transduced TCR-T58 T cells (red line) and TCR-T58 T cells transduced to express the native CD40L (blue line) were used as reference. TCR-T58 T cells transduced with CD40L:IgGFc:CD28 are depicted in orange, those with CD40L:Fil3:CD28 CSP are depicted in green and T cells with CD40L:CD28i CSP are depicted in purple. **(A)** Target cells were SK-Mel23 cells or SK-Mel23 that had been pre-treated with anti-CD40 antibody in a 1:11 concentration (clone HB14 pure functional grade, Miltenyi) before setting the co-culture to block the endogenous CD40 receptor. Histograms of CD40 expression on SK-Mel23 cells, line histogram depicts the specific staining for CD40 and the grey filled histogram is the unstained control. **(B)** Target cells were HEK293/Tyr/mock cells that had very low endogenous CD40 expression or were transduced to strongly express CD40 (HEK293/Tyr/CD40). Line histogram depicts the specific staining for CD40 and the grey filled histogram is the unstained control. This is one representative experiment of two.

After observing that the CD40L:CD28 CSPs displayed similar expression kinetic as the endogenous CD40L protein, and that upregulation is possible with CD3/CD28 activation, it was tested if the physiologic downregulation through CD40 receptor interaction that is well documented for the endogenous CD40L expression (30, 67), also applies to the CD40L:CD28 CSPs. The experimental set-up is depicted in **Figures 6A, B**. CD40L:CD28-transduced TCR-T58 T cells were co-cultured with the melanoma tumor cell line (SK-Mel23) (**Figure 6A**), which provides the activation stimulus through the TCR-peptide/MHC interaction (HLA-A2/tyrosinase, which is the ligand for TCR-T58) and expresses CD40 endogenously. The CD40L:CD28-transduced T cells were used freshly thawed when their CD40L:CD28 CSP expression level was absent, except for some residual expression of the CD40L:IgGFc:CD28 CSP. CD40L staining to detect CD40L:CD28 CSP surface expression was performed at the start of the co-culture, after 16 h, 24 h and 48 h. As depicted in the line graph of **Figure 6A**, induced surface expression of CD40L:CD28 CSPs was noticeable for the CD40L:IgGFc:CD28 with a steady incline until 48 h. A delayed and weak surface expression occurred for the CD40L native protein, the CD40L:Fil3:CD28 and CD40L:CD28i CSPs. Endogenous CD40L surface expression on mock T cells was not detected. All CD40L:CD28 CSPs showed strong induction of surface expression when the melanoma cell line was pretreated with CD40L antibody before it was used in the co-culture. These results suggested that CD40L:CD28 CSPs are upregulated by physiologic TCR activation through peptide/MHC interaction and subsequently downregulated by interaction with tumor cell-expressed CD40 receptor. Upregulation was achieved after 16 hours of co-culture, reaching a sustained increase expression until the 48 h time point in the presence of CD40 blockade.

In a second co-culture experiment (**Figure 6B**), HEK293 cells were used that endogenously express HLA-A2 and were transduced to stably express and present the tyrosinase antigen (HEK/Tyr cells). HEK/Tyr cells had very low endogenous CD40 expression and were, thus, additionally transduced to express CD40 (HEK/Tyr/CD40) or left transduced (HEK/Tyr/mock). In co-culture with HEK/Tyr/mock cells, which express only marginal CD40 receptor, strong induction of CD40L:IgGFc:CD28 as well as CD40L native was observed and lower induction of CD40L:Fil3:CD28 and CD40L:CD28i, which was comparable to the CD40L induction on T cells without CD40L:CD28 CSP transduction (mock). In contrast, using the HEK/Tyr/CD40 cells with strong CD40 expression, upregulation was only observed for the CD40L:IgGFc:CD28 CSP. Thus, surface expression of CD40L:CD28 CSPs induced by TCR-peptide/MHC interaction is counteracted by endogenous (SK-Mel23) or transgenic expression (HEK cells) of the CD40 receptor on target cells.

### B Cell Activation Induced by CD40L:CD28 CSPs Confirms ECD Biological Activity

**Figure 7:**
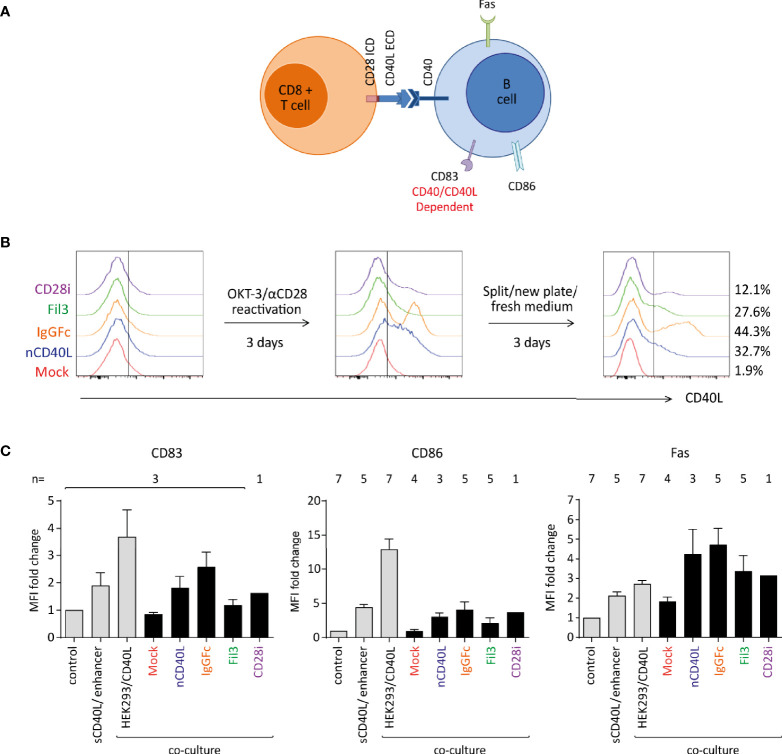
Biologic activity of the CD40L ECD within the CSPs using a B cell stimulation assay. Primary human T cells transduced with the CSPs were re-activated, collected after 6 days when endogenous CD40L expression on Mock-T cells was absent, and co-cultured with freshly isolated B cells at a 1:1 ratio. Co-cultures were harvested and analyzed by flow cytometry for B cell-specific surface activation markers CD83, CD86 and Fas. Three controls were included: i) a negative control consisting of B cells without T cells (unstimulated control), ii) a positive control using B cells stimulated with a soluble enhanced trimeric CD40L, and iii) B cells co-cultured with CD40L-expressing HEK293 cells. **(A)** Schematic diagram of the stimulation assay. **(B)** Histograms depicting the CD40L:CD28 CSP expression on the transduced T cells after thawing and anti-CD3/CD28 re-activation and culture for 6 days. **(C)** Graphs depicting the fold change of median fluorescence intensity (MFI) relative to control of CD83, CD86 and Fas on the surface of B cells after overnight co-culture with T cells transduced with CD40L:IgGFc:CD28 depicted in orange, CD40L:Fil3:CD28 CSP depicted in green and T cells with CD40L:CD28i CSP depicted in purple. Mock-transduced T cells (red) and T cells transduced to express the native CD40L (blue) were used as references. Controls are the first three bars distinguished by light grey color. Bars are the mean values of indicated number of independent experiments. Error bars are the standard deviation of the corresponding number of experiments n.

Having determined that the composite proteins can be expressed on the cell surface, assessing if the different domains were functionally incorporated into the chimeric CD40L:CD28 CSPs was the next step. The biologic activity of the CD40L ECD was tested in two systems using a B cell stimulation assay and DC activation. It is well described that B cells express the CD40 receptor. Upon interaction with the CD40 ligand (CD40L), B cells undergo activation, which can be detected by measuring key surface activation markers on the B cells like CD86, Fas and CD83 (**Figure 7A**). Upregulation of the three markers, CD83, CD86 and Fas, are linked to the CD40/CD40L interaction. CD86 and Fas expression might also be influenced by TCR/MHC interaction that occurs between T cells and B cells when the latter serve as APCs. CD83 has been reported to be induced on B cells *via* CD40 engagement independent of TCR/MHC binding (68). In our system, the CD40L:CD28 CSP-transduced T cells co-expressed the tyrosinase-specific TCR-T58. B cells were isolated from blood of healthy donors. No tyrosinase was present in the system excluding B cell activation through antigen.

T cells without and with CD40L:CD28 CSPs were thawed and re-activated for 6 days using CD3/CD28 antibody-coated 24 well plate (**Figure 7B**) to re-induce CD40L:CD28 CSP expression. T cells were harvested on day 6 when CD40L:CD28 CSP levels were detectable, but the mock T cells were CD40L negative (red histograms), indicating absence of endogenous CD40L that would conceal effects of the CD40L:CD28 CSPs (**Figure 7B**).

To assess B cell activation, T cells were co-cultured with freshly isolated B cells overnight (12 h) at a 1:1 ratio followed by analysis of surface activation markers on the B cell population by flow cytometry (**Figure 7C**). It was observed that CD83, CD86 as well as Fas were induced on B cells that were co-cultured with T cells expressing the CD40L:CD28 CSPs or the native CD40L compared to the co-culture with mock T cells that did not express any CD40L construct or control B cells without T cell co-culture (control). Gradual levels of activation marker expression on B cells were observed depending on the variant of the CD40L:CD28 CSP expressed by the T cells, with highest expression of activation markers achieved with T cells expressing CD40L:IgGFc:CD28, followed by native CD40L, CD40L:CD28i and lowest CD40L:Fil3:CD28. As reference, two different positive controls were included to stimulate the B cell population: one control setting used a commercial soluble CD40L agonist combined with an enhancer to promote CD40L trimerization and, hence, B cell activation. The other control was a co-culture with HEK293/CD40L cells that stably expressed high levels of native CD40L. The sCD40L/enhancer agonist achieved induction of activation markers on B cells similar to the T cell co-culture with CD40L:CD28 CSP-engineered T cells. Highest levels of B cell activation were achieved with the HEK293/CD40L cells.

The observed effects on B cells provide proof of concept that the CD40L ECD within the CSPs is exerting biologic activity with efficiencies matching the surface expression level of each CD40L:CD28 variant.

### CD40L:CD28 CSP-Expressing T Cells Induce DC Maturation and a Pro-Inflammatory Secretome in Tumor-Conditioned ercDCs

**Figure 8:**
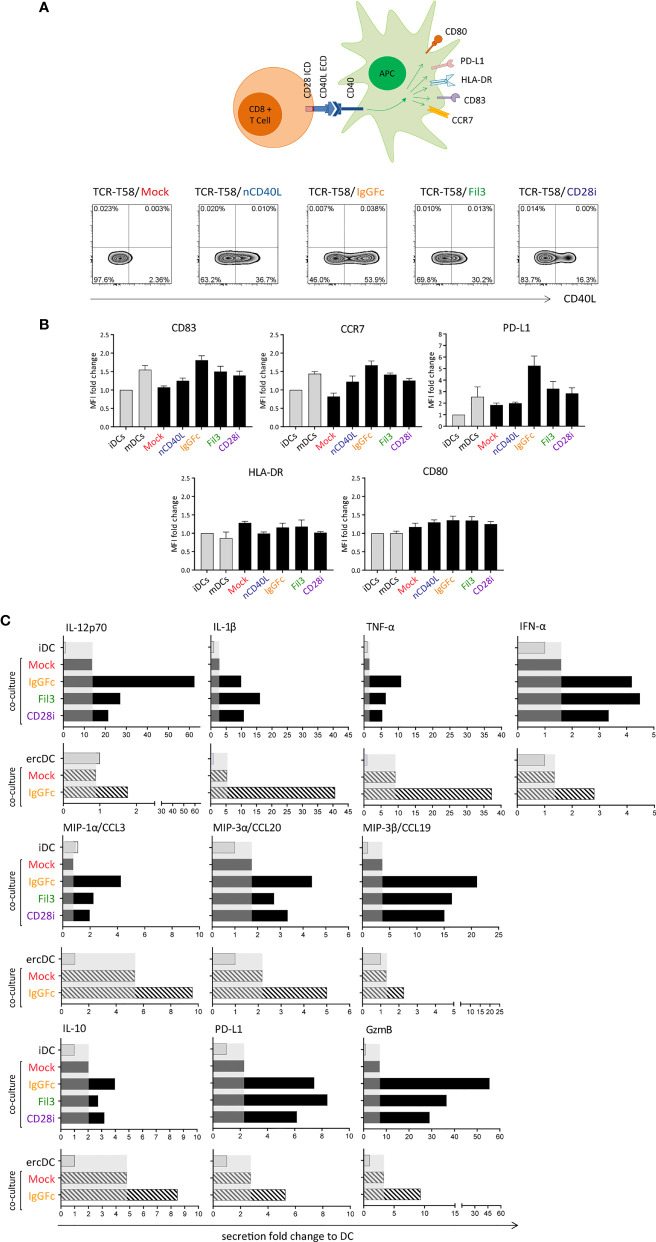
T cells expressing the CD40L:CD28 CSPs can activate dendritic cells and induce a pro-inflammatory secretome in tumor-conditioned ercDCs through CD40/CD40L interaction. **(A)** Schematic drawing of the DC maturation assay. TCR-T58 T cells expressing the different CSPs were thawed and re-activated to induce CSP expression as described in **Figure 7**. Co-cultures used immature dendritic cells (iDCs) or ercDCs. Negative control consisted of iDCs without T cells. Positive control consisted of mature DCs (mDCs). Density plots depicting the CD40L:CD28 CSP expression on the transduced T cells after re-activation. **(B)** Graphs depict the fold change of mean fluorescence intensity (MFI) relative to control of CD83, CCR7, PD-L1, HLA-DR and CD80 surface expression on DCs after overnight co-culture with CSP-expressing T58 T cells. Controls correspond to the first two bars distinguished by light colors, respectively. Bars are the mean values of 5 independent experiments. Error bars are the standard deviation of the 5 experiments. **(C)** Chemokine and cytokine secretion of iDCs and ercDCs after co-culture with T cells (measured using 45Plex bead array, representing thousands of individual bead measurements). Bars are the fold change in secretion relative to DC without T cell co-culture. Shaded area indicates the effect induced by Mock-T cells without CSP. Black bars indicate secretion by iDC, grey bars depict the secretion by ercDCs. ercDCs are tumor-conditioned DCs.

In the DC maturation assay, immature DCs (iDCs) were generated *in vitro* following a 7 day protocol (51), and the upregulation of CD83, CCR7, PD-L1, HLA-DR and CD80 was assessed after T cell co-culture (**Figure 8A**). To assess effects on DC maturation, *in vitro* generated iDCs were co-cultured with TCR-T58 T cells without or with CD40L:CD28 CSP expression (**Figure 8A**) overnight (12 h) in a 1:1 ratio. iDCs without T cells served as a negative control and DCs maturated with Jonuleit cytokine cocktail were used as a positive control. DCs were from HLA-A2 negative donors, thus providing no tgTCR-specific stimulation. In **Figure 8B**, the expression of DC maturation markers after each culture condition is depicted as the mean fluorescence intensity. For CD83, CCR7 and PD-L1, the expression levels were highest after co-culture with T cells expressing the CD40L:IgGFc:CD28, closely similar to levels observed on mDC. T cells expressing CD40L:Fil3:CD28 or CD40L:CD28i still induced levels above those achieved with Mock T cells, or T cells with native CD40L, or iDCs that were not stimulated at all. For HLA-DR and CD80 markers, no significant difference was seen between the different culture conditions.

In addition to effects on surface markers, CD40L:CD28-expressing T cells also caused changes in secreted cytokines and chemokines in co-cultures with DCs. These included augmented secretion of IL-12p70, IL-10, pro-inflammatory IL-1β, TNF, chemokines MIP-1α (CCL3), MIP-3α (CCL20) and MIP-3β (CCL19) as well as the cytotoxic protein granzyme B (gzmB) (**Figure 8C**). PD-L1 was also induced consistent with the observed upregulation on the cell surface. No changes were seen for Th2 cytokines (IL-4, IL-5, IL-13), IL-17, IL-33, IL-15, eotaxin and fractalkine (CX3CR1), TRAIL, PDGF or EGF (not shown).

In the tumor milieu, myeloid cells adopt TME-associated phenotypes that might be associated with tumor promotion and immune cell inhibition. In RCC, we previously reported a uniquely polarized myeloid subset, which we called ercDC (*enriched in renal cell carcinoma* DC) (52, 69). ercDC were found to exhibit protumorigenic and immune inhibitory features and a high prevalence in tumor tissue correlated with poor survival. Here we used *in vitro* generated surrogate ercDCs (52) for co-culture and observed that CD40L:CD28-expressing T cells induced a similar pro-inflammatory and chemotactic secretome as observed for *in vitro* generated iDCs.

### The CD28 Signaling Domain in CD40L:CD28 CSPs Is Biologically Active Evidenced by Induction of the AKT Pathway

In a next step it was determined whether the CD28 ICD was capable to deliver a proper co-stimulation signal to the T cells. The CD28 co-stimulatory pathway starts with phosphatidylinositol 3′-kinase (PI3K) leading to phosphorylation of the protein Kinase B, also known as AKT, followed by downstream phosphorylation of the mammalian target of rapamycin (mTOR) molecule. Ribosomal protein S6 (RPS6) is then a downstream target of mTOR activation (70, 71).

The functionality of the CD28 ICD within our CD40L:CD28 CSPs was tested by analyzing the phosphorylation of AKT, mTOR and RPS6 in CD40L:CD28 CSP-transduced tyrosinase-specific TCR-T58 T cells after stimulation with HEK293/Tyr/CD40 cells, which can trigger the T58-TCR through HLA-A2/Tyr ligands and the CD40L:CD28 CSPs through CD40 expression. Before setting up the stimulation co-culture, T cells were deprived of IL-2 for 4 hours to reduce the level of constitutive AKT activation. Subsequently, the starved T cells were co-cultured with the HEH293/Tyr/CD40 cells for 30 minutes, which was previously determined as the optimal time for AKT activation to reach its peak (72, 73).

**Figure 9:**
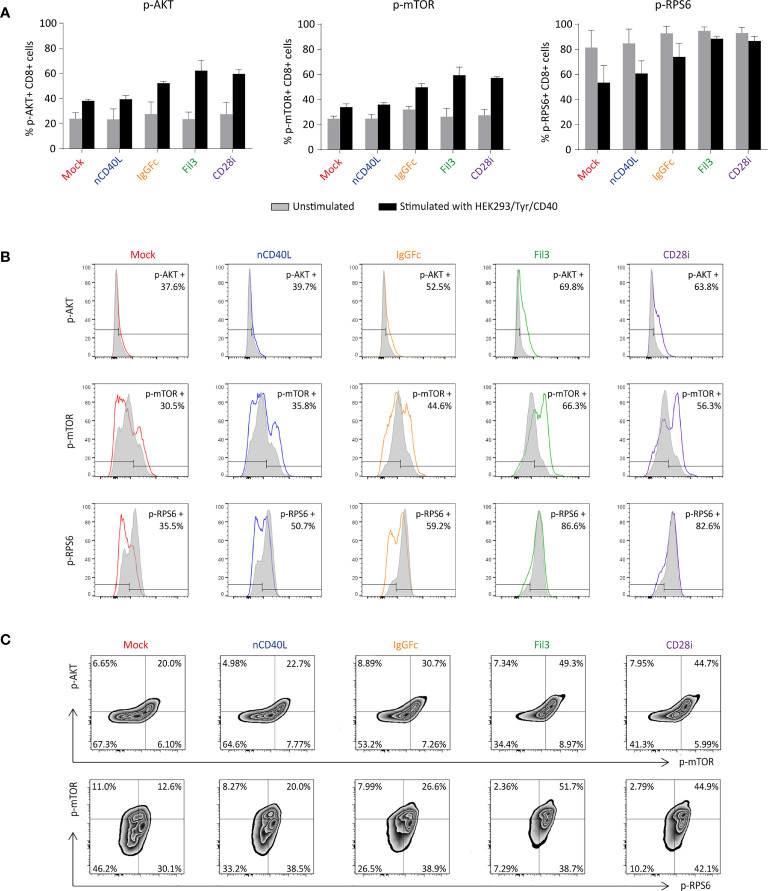
Biological activity of the CD28 ICD within the CSPs by measuring the phosphorylation of AKT, mTOR and RPS6. CD40L:CD28 CSP-expressing TCR-T58 T cells were thawed and re-activated to induce CSP expression as described in **Figure 7**. On day 6, they were stimulated with HEK293/Tyr/CD40 for 30 min. Unstimulated T cells were used as a negative control. **(A)** Graphs depict the percentage of phosphorylated p-AKT, p-mTOR and p-RPS6 in T cells after 30 minutes co-culture, grey bars correspond to the unstimulated controls and black bars correspond to the stimulated T cells. Bars are the mean values of the percentages of three independent experiments. The error bars are the standard deviation. **(B)** Histograms depicting the fluorescence intensity of each phosphorylated protein in T cells expressing the different CSPs; unstimulated (grey histogram) and after stimulation (colored histograms). **(C)** Density plots showing the correlation between p-AKT with p-mTOR and p-mTOR with p-RPS6 in T cells expressing the different CSPs. Mock-transduced T cells (red) and T cells transduced to express the native CD40L (blue) were used as references. T cells transduced with CD40L:IgGFc:CD28 are depicted in orange, those with CD40L:Fil3:CD28 CSP are depicted in green and T cells with CD40L:CD28i CSP are depicted in purple.

After 30 minutes of stimulation, phosphorylation of AKT protein was increased in CSP-transduced T cells in comparison with the Mock T cells without CSP or T cells carrying the native CD40L that does not contain the CD28 signaling domain, or the unstimulated T cells (**Figure 9A**, left graph). Similar results were detected for the phosphorylation of the mTOR and RPS6 proteins (**Figure 9A**, middle and right graphs), suggesting that the CD40L:CD28 CSPs activated the AKT pathway.

Induction of phosphorylation was strongest in T cells that expressed the CD40L:Fil3:CD28 or the CD40L:CD28i compared to T cells with the CD40L:IgGFc:CD28. The differences between the three CD40L:CD28 CSPs can be appreciated in the histogram display, showing increased MFI of phosphorylated AKT and mTOR relative to the unstimulated T cells when the T cells expressed the CSPs (colored histogram), while the MFI in Mock and native CD40L-expressing T cells was similar to unstimulated control (**Figure 9B**). The pattern of the phosphorylated RPS6 (p-RPS6) differed from those of AKT and mTOR showing lower MFI in stimulated T cells that expressed no CSP or the native CD40L protein compared with the unstimulated control, while phosphorylation was higher in stimulated T cells expressing the CD40L:Fil3:CD28 or the CD40L:CD28i CSP, reaching levels of the unstimulated T cells.

Finally, the correlation of phosphorylation of the CD28 downstream signaling proteins was depicted by plotting the p-AKT against p-mTOR and p-mTOR against p-RPS6 (**Figure 9C**). The percentages of double-positive populations, corresponding to T cells that had phosphorylated both AKT and mTOR or mTOR plus RPS6, was notably higher in T cells that were transduced with the CD40L:CD28 CSPs compared to Mock T cells without CSP or T cells transduced with the native CD40L that does not contain a CD28 signaling domain.

### CD40L:CD28 CSPs Improve T Cell Function

After the biologic activity of both domains of the CD40L:CD28 CSPs was demonstrated, it was tested if the CD28 signaling provided by the CSPs was able to improve T cell effector function, namely cytokine secretion and cytotoxicity.

**Figure 10:**
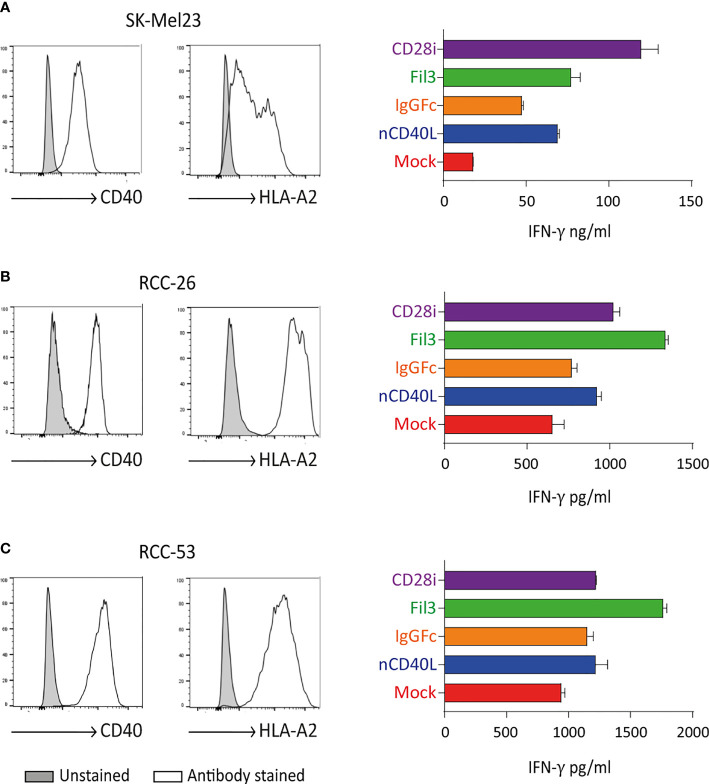
CD40L:CD28 CSPs improve T cell cytokine secretion. TCR-T58 tyrosinase-specific T cells and TCR53 RCC-specific T cells expressing the CD40L:CD28 CSPs were thawed and used immediately without re-activation in co-cultures at a T cell to target cell ratios of 1:10 with tyrosinase-positive target cells SK-Mel23 for TCR-T58 T cells **(A)**, and RCC-26 and RCC-53 RCC tumor cell lines for TCR53 T cells **(B, C)**. All target cells expressed CD40 and HLA-A2 endogenously (shown as histograms). IFN-ү was measured in supernatants of co-cultures by ELISA. Shown are mean values of duplicates from one representative experiment repeated 2 times. Error bars are the standard deviation.

Tyrosinase antigen-specific TCR-T58 T cells transduced to express the different CD40L:CD28 CSPs secreted more IFN-γ when stimulated with SK-Mel23 cells (tyrosinase/HLA-A2 and CD40 positive) compared to TCR-T58/mock T cells without CD40L:CD28 CSP (**Figure 10A**). Similar results were observed when CD40L:CD28 CSP-transduced RCC-specific TCR53 T cells were co-cultured with CD40 positive RCC-26 and RCC-53 cell lines (**Figures 10B, C**).

Among the different CSP formats, the CD40L:IgGFc:CD28 (orange) had the lowest effect on cytokine secretion, while the CD40L:CD28i (purple) and the CD40L:Fil3:CD28 enhanced the cytokine secretion more strongly. This is of note, since it contrasts with the expression level of the CSPs on the T cells where the CD40L:IgGFc:CD28 consistently had highest levels and CD40L:Fil3:CD28 was lowest (see for example **Figure 3**).

**Figure 11:**
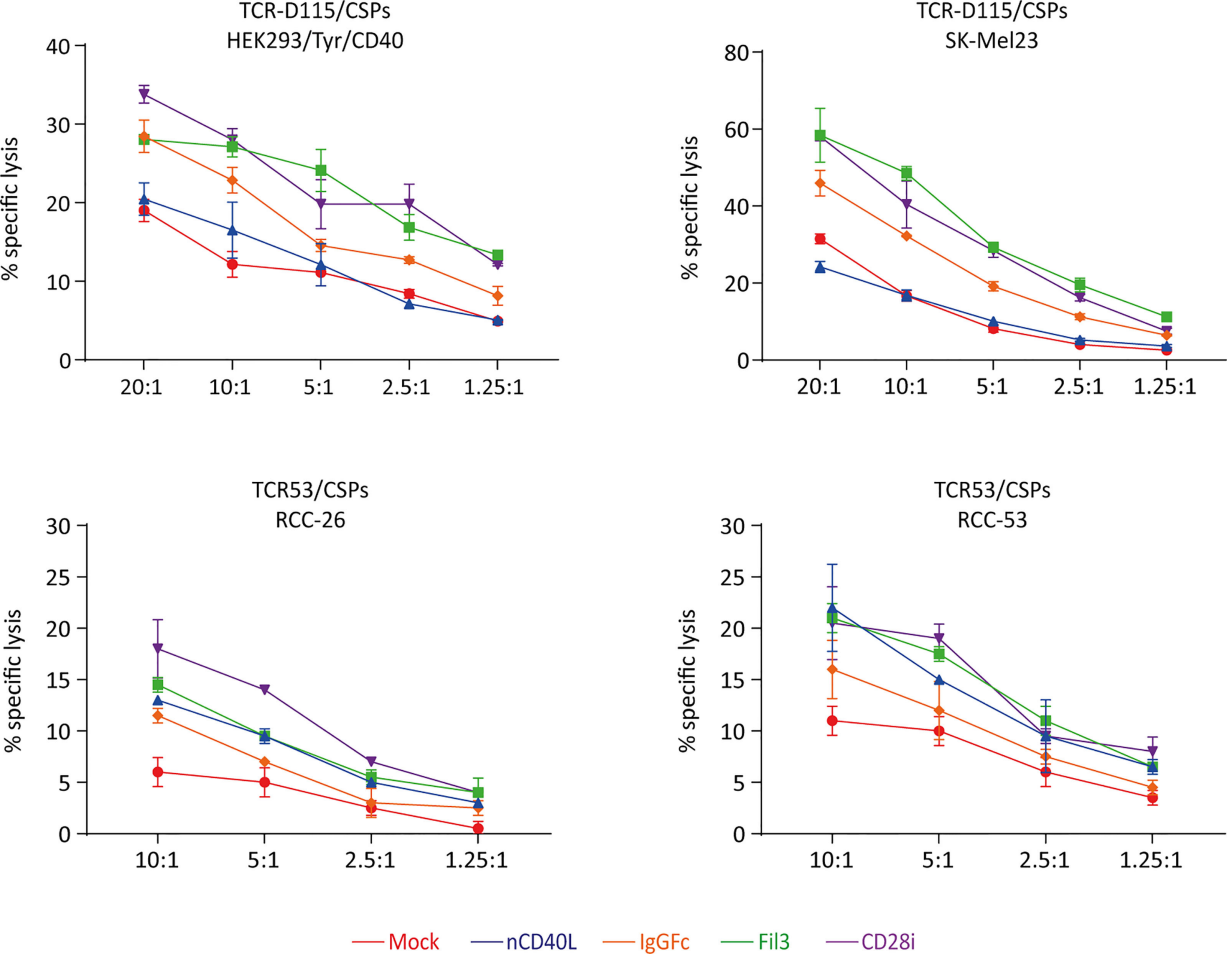
CD40L:CD28 CSPs improve cytolytic activity of antigen-specific T cells. TCR-D115 tyrosinase-specific T cells and TCR53 RCC-specific T cells expressing the CD40L:CD28 CSPs were thawed and used immediately without re-activation in a 4h chromium release assay at indicated T cell to target cell ratio with tyrosinase-positive target cells SK-Mel23 and HEK293/Tyr/CD40 for TCR-D115 T cells, and RCC-26 and RCC-53 RCC tumor cell lines for TCR53 T cells. Shown are mean values of duplicates from one representative experiment. Error bars show the standard deviation. All target cells expressed CD40 and HLA-A2 (see histograms **Figures 6**, **10**).

Target cell killing using tyrosinase-specific TCR-D115 T cells and RCC-specific TCR53 T cells showed that T cells expressing the CD40L:CD28 CSPs executed higher target lysis compared to the T cells without CSPs (**Figure 11**). As noted before for the cytokine secretion, CD40L:CD28i (purple) and CD40L:Fil3:CD28 (green) CSPs supported T cells more strongly than the CD40:IgGFc : CD28 (orange line).

Of note, anticipating future clinical application, T cells in the functional assays were used freshly thawed without *in vitro* re-activation, thus the starting expression of the CSPs was low. The results of both the cytokine secretion and the target cell killing suggest that positive effects of the CD40L:CD28 CSPs on two central effector functions can be expected despite low CSP expression.

## Discussion

In this work, a novel CSP design was explored combining the extracellular domain of the CD40L protein with the intracellular domain of the CD28 co-stimulatory receptor. With this combination, a double strike against tumor is hypothesized consisting of boosting T cell activity against tumor cells and additionally attacking tumor stroma. The extracellular CD40L domain of the CSP is key to this double action approach since its receptor, CD40, is aberrantly expressed by many tumor cells, including RCC and melanoma, and is also expressed on tumor stroma, including antigen presenting cells, tumor-associated macrophages (45–48, 52) and tumor endothelial cells (40–42) (see **Figure 1**).

The chimeric design of CD40L with CD28 required combining domains of a type II (CD40L) and type I (CD28) membrane protein. The principles of this type of combination are not yet clearly defined (23) and represent a challenge in terms of achieving surface expression and biological function. For that reason, three different protein structures were engineered and tested with the aim of elucidating the proposed mechanisms of action of a CD40L:CD28 CSP.

Two approaches created a chimeric type I transmembrane protein. For this, the sequence of the soluble extracellular domain of CD40L (sCD40L) was inverted and connected to the transmembrane plus intracellular CD28 signaling sequence *via* spacer and Glycine/Serine linker, as commonly used in the CAR design (74, 75). Two different spacers were used, the IgG1Fc and Fil3. The IgG1Fc spacer aims to give stability and to increase surface expression to the resulting chimeric protein through dimerization (61). The Fil3 is the third Ig-like repetition of the cytoskeletal structural Filamin protein and was selected to address the concern of potential high surface polymerization that the IgG1Fc spacer might cause and that might impact the protein functionality. The Fil3 fragment lacks polymerization domains, tends to be more flexible and is considerably shorter than the IgG1Fc spacer (62), conferring an interesting variation towards the construction of a functional CD40L:CD28 CSP.

For the third CD40L:CD28 design, the extracellular and transmembrane domains of CD40L were kept in the orientation of the native CD40L protein and were directly linked to the inverted intracellular co-stimulatory domain of the CD28 protein (CD28i). The final structure of the third CSP CD40L:CD28i resembled a type II membrane protein and had a similar size to the endogenous CD40L protein. This approach offered a design with less structural modifications and suggested a more natural interaction with the CD40 receptor.

After retroviral transduction, the CD28i and the IgGFc constructs were highest expressed, while the Fil3 construct was in comparison poorly expressed. Although retroviral transduction is a process that should result in stable expression of transduced sequences, this was not the case for the CD40L:CD28 CSPs. Over 13 days of culture, expression of all constructs gradually decreased. The Fil3 CSP was almost undetectable after 6 days, the nCD40L was lost at day 10, the CD28i CSP at day 13, and the IgG1Fc CSP remained detectable by day 13 at 52% expression compared to the initial level.

Culture conditions, T cell activation using anti-CD3 and anti-CD28, and specific activation signals delivered by TCR-MHC/peptide interaction were able to induce re-expression of the CSP on T cells and the presence of the CD40 receptor on the target cells caused its subsequent downmodulation.

It is known that endogenous CD40L expression on CD4 T cells exhibits a continuous cycle of downregulation and re-expression upon interaction with CD40 expressing B cells, with this being dependent on the presence of antigen (66, 76, 77). At the same time, the re-expression could be stabilized by provision of CD28 co-stimulation, which might become more pronounced with time (65). Regulation of CD40L mRNA stability during T cell activation is suggested as one mechanism contributing to the dynamic of the endogenous CD40L expression (78). A similar regulation might explain the dynamic of our nCD40L protein, but it shouldn´t be the case for the CSPs, as these have significant structural differences from the CD40L protein. Since it still occurred for our constructs, a cell activation associated factor or a post-translational degradation (79) might be regulating the CSP expression. In an effort to determine if transcription, transport defect, or internalization could explain the surface dynamics of our CD40L:CD28 CSPs, RNA levels and total protein were quantified by qPCR and western blot. However, the results were not conclusive enough to develop a compelling explanation. Results by Higham et al. (45) depict a similar surface downregulation of retrovirally transduced CD40L on CD8 T cells, while Thy1.1 expressed from the same retroviral vector was stably maintained. Deletion of the terminal 13 AA residues of CD40L prolonged surface expression time but did not prevent eventual loss of surface expression. Higham et al. suggested that further engineering processes could help overcome the transient nature of expression despite retroviral transduction. However, details were not specified.

In our study, similar to the one described above (45), the CD40L:CD28 CSP expression was clearly related to antigen encounter: Upon TCR ligation through tumor-expressed peptide/MHC the CSPs were re-expressed and once the tumor cells were eradicated, the CSPs underwent downmodulation until T cells were again activated. Such expression kinetic with the upregulation following T cell stimulation might be advantageous were it to happen under *in vivo* circumstances. A beneficial co-stimulatory effect might, thereby, occur timely coupled with the recognition of tumor cells through the TCR-MHC interaction, providing help for improved T cell functionality for as long as tumor cells are present and the antitumoral response is required. The “inbuilt” stopp of the T cell support after removal of the activator (i.e. antigen-positive tumor or infected cell) could prevent undesired over-activation of the immune system through the CD40L domain of the CSP.

In terms of biological activity, the extracellular CD40L domain of the CSPs was able to functionally interact with B cells and DCs making them potentially more effective antigen presenting cells. The extent of effect correlated to the expression level on the T cell surface with the IgGFc construct showing highest effects followed by the CD28i CSP and Fil3 CSP. Changes included the upregulation of MHC class II and co-stimulatory molecules (CD80/CD86) as well as maturation markers, like CD83, CCR7 and PD-L1 as was expected from the literature (31, 80, 81).

Moreover, T cells that were equipped with CD40:CD28 CSPs stimulated iDCs to secrete pivotal cytokines and chemokines for lymphocyte recruitment and T cell stimulation. These included IL-12p70, pro-inflammatory IL-1β, and TNF, as well as MIP-3α (CCL20) and MIP-3β (CCL19), which are chemotactic for lymphocytes and DCs, and IFN-α, which is known to activate antigen presentation for T cell-mediated tumor cell recognition (82). Notably, CSP-associated secretion of granzyme B was observed in the absence of cognate TCR-peptide/MHC ligand interaction. Further investigation is required to address if the antigen-independent activation of the CD40L/CD40 pathway will allow antigen-independent killing of stroma cells eliminating the tumor-supporting environment with subsequently better tumor control.

CD40L:CD28-transgenic T cells induced similar effects on ercDCs, which are tumor-conditioned myeloid cells found in human RCC (52, 69). ErcDCs are described to display pro-tumorigenic and immune inhibitory features. A high prevalence in tumor tissue was found to correlate with poor survival. ErcDC were activated by CD40L:CD28 CSP-expressing T cells to secrete IL-12p70, lymphocyte recruiting chemokines, IL-1β, TNF, as well as IL-10. Previous studies have reported the requirement of CD40/CD40L activation of tumor DCs for effective antitumor T cell therapy (45, 46). Recent studies highlight the importance of IL-1ß secretion by intratumoral DCs for the maintenance of CD8 effector T cells in the TME (83) and IL-10 for re-programming exhausted CD8 T cells (84). Upregulation of PD-L1 on DCs was seen after contact with CD40L:CD28 CSP-expressing T cells suggesting that reprogramming of tumor-conditioned myeloid cells (85) together with checkpoint inhibition could be the next step forward to improve cancer immunotherapy (86, 87). The here reported activation of a pro-inflammatory secretome in TME-conditioned ercDCs is an encouraging sign that CD40L:CD28 CSPs might be able to promote myeloid cell repolarization in the TME.

Collectively, the observed effects on B cells and DCs confirm the functionality of the CD40L extracelluar domain and, moreover, provide support for our proposed hypothetical mode of action of CD40L:CD28 CSP-expressing T cells where tumor-resident APCs can receive the benefit of stimulation through the CSP to rescue their activity in the TME.

For the CD28 domain, functionality was demonstrated by augmented phosphorylation of the AKT protein and downstream AKT targets, mTOR and RPS6 proteins, after co-culturing the CSP-transduced T cells with antigen-specific CD40-positive target cells. The strength of the effect was inversely associated with the level of CSP surface expression on T cells: The most highly expressed IgGFc CSP evoked weakest activation of the phosphorylation cascade while the Fil3 CSP, although being low expressed, and CD28i CSP (despite its inverted orientation) had stronger effects.

The presence of the CD28 signaling domain in the CSP conferred improved cytokine secretion and antigen-specific cytotoxicity to tgTCR T cells when in contact with target cells expressing specific antigens and the CD40 receptor. The extent to which T cell function was enhanced correlated to the level of AKT pathway activation which was best achieved through the CD40L:Fil3 and CD40L:CD28i CSPs. High surface expression was apparently not determining the signaling quality and cis-effect on T cell functionality. Explanations for this observation remain speculative. The CD40L-CSP containing the IgGFc spacer is the longest one of the three CD40L:CD28 CSPs. The longer distance of the extracellular domain from the membrane surface might impact the signaling outcome as has been seen in CAR designs (88, 89). The CSPs with the Fil3 spacer and the CD28inverse domain are close in size to the native CD40L protein and, apparently, are better suited to facilitate signaling and effector functions in T cells. Of note is that the T cells used in functional assays were not pre-activated but used freshly thawed as it would be done in the clinical setting of ACT. Thus, the CSPs were barely expressed on the T cell surface. Nevertheless, CD40L:CD28 CSP-engineered T cells received signaling with supportive outcome regarding effector function within the short time frame of 4 hours (killing assay) and 24-48 hours (cytokine secretion). The TCR-MHC interaction and the presence of the CD40 receptor on the target cells seem to quickly activate CSP expression and T cell supportive signaling events.

CD40 agonists are explored in clinical trials aiming at moderating macrophages and stroma influences as well as augmenting response to chemotherapy and immunotherapy (90–92). Concerns of systemic side effects limit broad utilization of these agents, thus, intratumoral delivery strategies are being explored (93). Our CSP design offers a solution to this hurdle by securing the CD40L to the T cell surface and delivering it to the TME through T cell infiltration. Overall, combining CD28 and CD40/CD40L effects within a chimeric CD40L:CD28 design shows promise to deliver CD40 activation to the TME together with improved T cell functionality for tumor attack.

## Author's Note

The results are part of a doctoral thesis submitted to the Ludwig Maximilian University of Munich.

## Data Availability Statement

The original contributions presented in the study are included in the article/**Supplementary Material**. Further inquiries can be directed to the corresponding author.

## Ethics Statement

The studies involving human participants were reviewed and approved by Ludwig Maximilians University, Ethical Committee. The patients/participants provided their written informed consent to participate in this study.

## Author Contributions

Conception and design, LO-C, AM, BH, GP and EN. Development of methodology, LO-C, AM, and EN. Acquisition of data, LO-C. Analysis and interpretation of data, LO-C, AM, and EN. Writing of the manuscript, LO-C and EN. Review, and/or revision of the manuscript, LO-C, AM, GP, BH, and EN. All authors contributed to the article and approved the submitted version.

## Funding

The work was supported through funding Deutsche Krebshilfe, SFB-TR36, Erich & Gertrud Roggenbuck Stiftung and DAAD-CONACyT scholarship cooperation program.

## Conflict of Interest

EN declares financial relationship due to patent WO2017/162797.

The remaining author(s) declare(s) that the research was conducted in the absence of any commercial or financial relationships that could be construed as a potential conflict of interest.

## Publisher’s Note

All claims expressed in this article are solely those of the authors and do not necessarily represent those of their affiliated organizations, or those of the publisher, the editors and the reviewers. Any product that may be evaluated in this article, or claim that may be made by its manufacturer, is not guaranteed or endorsed by the publisher.
