# Supplementary material for: Double Strike Approach for Tumor Attack: Engineering T Cells Using a CD40L:CD28 Chimeric Co-Stimulatory Switch Protein for Enhanced Tumor Targeting in Adoptive Cell Therapy
Source: Front Immunol. 2021 Nov 29;12:750478. doi: 10.3389/fimmu.2021.750478 (PMC8666660; doi:10.3389/fimmu.2021.750478)
Supplement: Supplementary file 1 [file DataSheet_1.docx]

Double strike approach for tumor attack: Engineering T cells using a CD40L:CD28 chimeric co-stimulatory switch protein for enhanced tumor targeting in adoptive cell therapy.

Luis Felipe Olguín-Contreras^1^, Anna N. Mendler^1^, Grzegorz Popowicz^2^, Bin Hu ^1^, Elfriede Noessner^1,3^

^1^Institute of Molecular Immunology, Helmholtz Center Munich, Munich, Germany, ^2^Institute of Structural Biology, Helmholtz Center Munich, Munich, Germany, ^3^Immunoanalytics Research Group - Tissue control of Immunocytes, Helmholtz Center Munich, Munich, Germany.

*** Correspondence:**Elfriede Noessner: Immunoanalytics-Tissue control of Immunocytes, Helmholtz Center Munich, Munich, Germany, Feodor-Lynen Str. 21; +498931871303; noessner@helmholtz-muenchen.de

**Supplementary Material:**

**
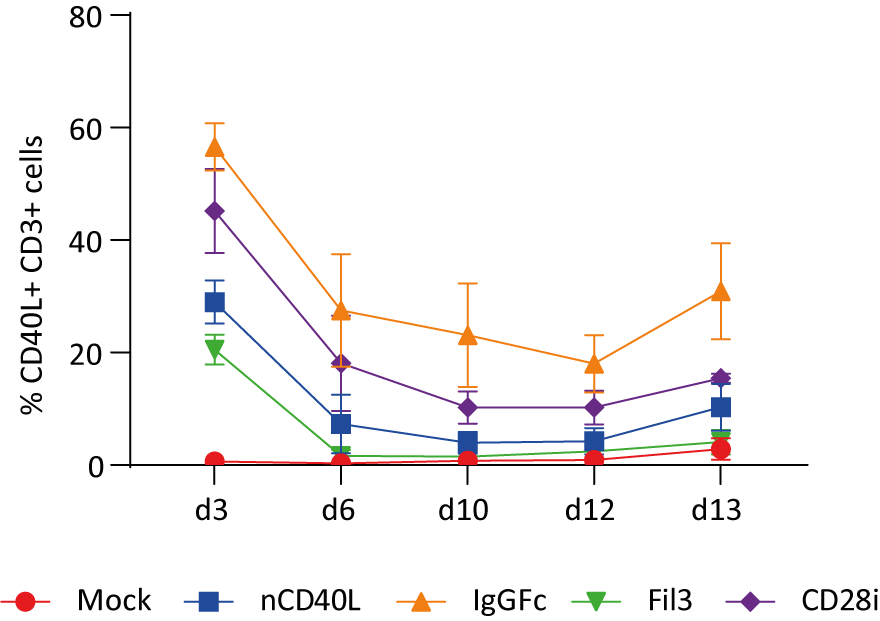
**

**Supplementary Figure 1: CD40L:CD28 CSP surface expression kinetic in PBLs after retroviral transduction**

Human primary T cells were retrovirally transduced with pMP71 encoding the CD40L:CD28 sequences and CSP surface expression was measured by flow cytometry on days 3, 6, 10, 12 and 13 after transduction using anti-CD40L-PE antibody (clone 89-76, eBioscience). The percentage of CD40L positive cells within gated live, single, CD3+ populations was determined on indicated days after retroviral transduction. Shown is the summary graph of 5 independent experiments. Symbols are the mean of gated percentages ±SEM. Mock-transduced T cells were used as negative control (red line), T cells transduced with the native CD40L (nCD40L) protein were used as expression reference and are depicted in blue, CD40L:IgGFc:CD28 CSP is depicted in orange, CD40L:Fil3:CD28 CSP is depicted in green and CD40L:CD28i CSP is depicted in purple.

**
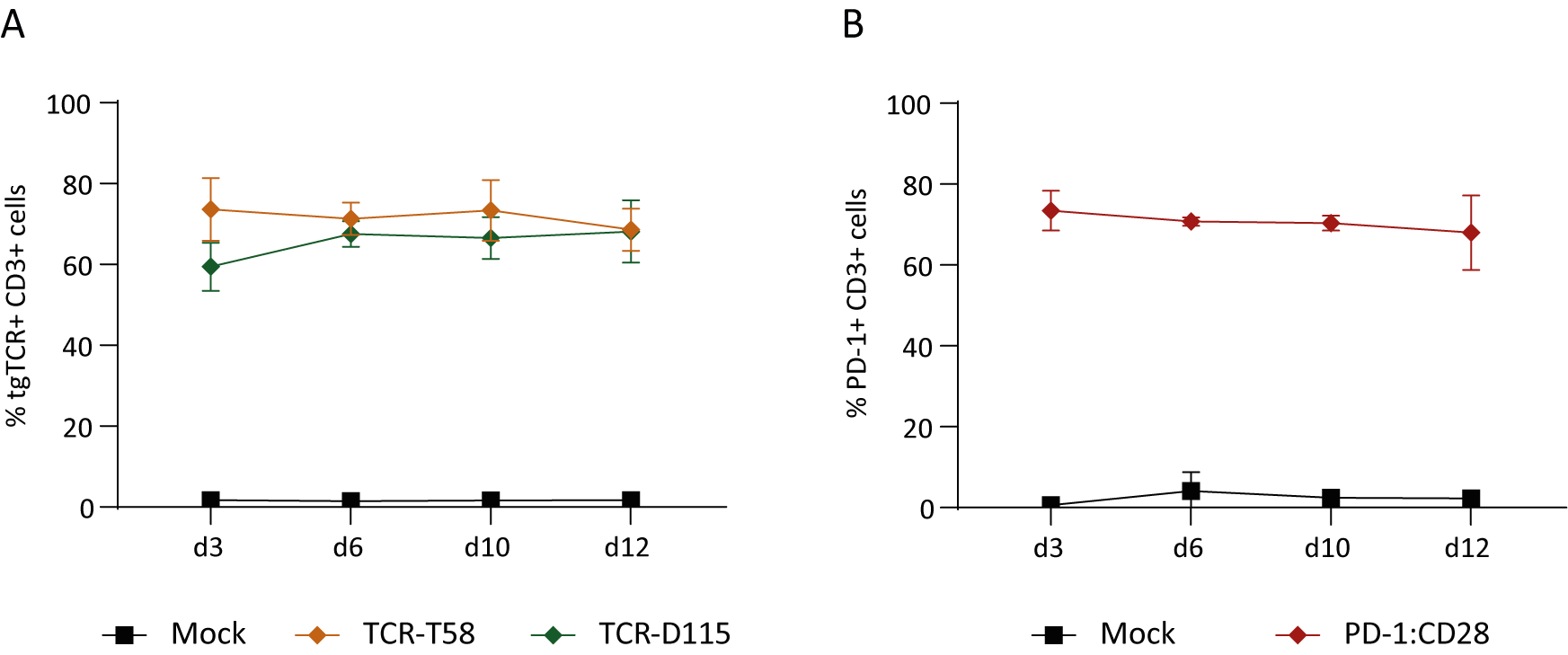
**

**Supplementary Figure 2: Expression dynamic of TCRs and PD-1:CD28 CSP constructs after retroviral transduction in T cells.**

Human primary T cells were retrovirally transduced with pMP71 encoding human TCR-T58, TCR-D115 or PD-1:CD28 CSP (Schlenker et al. 2017). Mock-transduced T cells were used as negative control (black). **(A)** Surface expression of transgenic (tg)TCR on gated live, single CD3+ T cells was assessed by flow cytometry using anti-mouse TCRβ-constant region (mTCR)-PB (H57-59, BioLegend) on days 3, 6, 10 and 12 after transduction. Symbols are the mean of percentages of tgTCR+ T cells ±SEM of 3 independent experiments. **(B)** Surface expression of PD-1:CD28 CSP assessed by flow cytometry using anti-PD-1 antibody (PE, MIH4, eBioscience). Symbols are the mean of percentages of tgPD-1:CD28+ T cells ±SEM of 4 independent experiments.
